# Growth Hormone Promotes Hair Cell Regeneration in the Zebrafish (*Danio rerio*) Inner Ear following Acoustic Trauma

**DOI:** 10.1371/journal.pone.0028372

**Published:** 2011-11-30

**Authors:** Huifang Sun, Chia-Hui Lin, Michael E. Smith

**Affiliations:** Department of Biology and Biotechnology Center, Western Kentucky University, Bowling Green, Kentucky, United States of America; Federal University of Rio de Janeiro, Brazil

## Abstract

**Background:**

Previous microarray analysis showed that growth hormone (GH) was significantly upregulated following acoustic trauma in the zebrafish (*Danio rerio*) ear suggesting that GH may play an important role in the process of auditory hair cell regeneration. Our objective was to examine the effects of exogenous and endogenous GH on zebrafish inner ear epithelia following acoustic trauma.

**Methodology/Principal Findings:**

We induced auditory hair cell damage by exposing zebrafish to acoustic overstimulation. Fish were then injected intraperitoneally with either carp GH or buffer, and placed in a recovery tank for either one or two days. Phalloidin-, bromodeoxyuridine (BrdU)-, and TUNEL-labeling were used to examine hair cell densities, cell proliferation, and apoptosis, respectively. Two days post-trauma, saccular hair cell densities in GH-treated fish were similar to that of baseline controls, whereas buffer-injected fish showed significantly reduced densities of hair cell bundles. Cell proliferation was greater and apoptosis reduced in the saccules, lagenae, and utricles of GH-treated fish one day following trauma compared to controls. Fluorescent *in situ* hybridization (FISH) was used to examine the localization of GH mRNA in the zebrafish ear. At one day post-trauma, GH mRNA expression appeared to be localized perinuclearly around erythrocytes in the blood vessels of the inner ear epithelia. In order to examine the effects of endogenous GH on the process of cell proliferation in the ear, a GH antagonist was injected into zebrafish immediately following acoustic trauma, resulting in significantly decreased cell proliferation one day post-trauma in all three zebrafish inner ear end organs.

**Conclusions/Significance:**

Our results show that exogenous GH promotes post-trauma auditory hair cell regeneration in the zebrafish ear through stimulating proliferation and suppressing apoptosis, and that endogenous GH signals are present in the zebrafish ear during the process of auditory hair cell regeneration.

## Introduction

Sensory hair cells in the auditory and vestibular portions of the inner ear transduce mechanical signals into neural ones, and thus are essential for hearing and balance [1]–[4]. Hearing loss and balance degeneration caused by loss of hair cells are irreversible in humans since auditory and vestibular hair cells do not regenerate in adult mammals [5]–[7]. 

The production of new hair cells has been elicited in the cochlea of mammals by manipulating key molecules associated with cell proliferation. For example, genetically-engineered rodents carrying altered Rb1 or Atoh1 (Math1) alleles formed new functional auditory hair cells after damage [8]–[11]. Derived from Atoh1 (Math1) transgenic mice, embryonic and pluripotent cells are able to differentiate and proliferate into mechanosensitive hair cells in culture with the manipulation of other genes [12]. While hair cell regeneration can be induced with the gene technologies referenced above, such as homologous recombination, transgenic expression or adenoviral infections, it does not occur spontaneously in the adult mammalian cochlea.

In contrast, hair cell regeneration occurs spontaneously in birds, reptiles, amphibians and fishes following hair cell loss due to either acoustic or ototoxic trauma [13]–[19]. Understanding the process of hair cell regeneration in non-mammalian vertebrates may lead to potential therapeutic applications in humans. The zebrafish (*Danio rerio*) has become an important vertebrate model for examining embryogenesis, organ development, disease, and genetic defects [20]–[23], and more recently as a model of hair cell regeneration. Much of the work on zebrafish hair cell regeneration has focused on the lateral line. Zebrafish lateral line neuromast hair cells are able to regenerate completely within 72 hours of hair cell loss induced by ototoxic chemicals [24]. This hair cell regeneration can be achieved through either proliferation of supporting cells, some of which differentiate into hair cells, or direct transdifferentiation of supporting cells into hair cells [25].

Recently, hair cell regeneration has also been reported in the teleost inner ear following acoustic overexposure [17], [26]. Acoustic overexposure for 48 hours leads to increased apoptosis in the goldfish (*Carrasius auratus*) saccule immediately following the exposure, and partial recovery of hair cells after eight days [17]. Preceding hair cell regeneration, which starts to occur by seven days following acoustic trauma in the zebrafish saccule, mitosis peaks at two days following acoustic exposure [26]. This suggests that cellular proliferation is likely an important mechanism by which zebrafish auditory hair cells regenerate, as is the case in hair cell regeneration in the avian ear [27].

Previous microarray analysis of zebrafish ear tissues that had experienced acoustic trauma identified potential genes involved in auditory hair cell regeneration. The gene that was most dramatically upregulated (64-fold) two days following sound exposure was growth hormone (*gh1*, *Danio rerio*) [28]. This coincides with the timing of cell proliferation in the zebrafish saccule following acoustic trauma, indicating the possible regulatory effects of GH during hair cell regeneration [26].

GH is a commonly known secretory protein of the cytokine superfamily of polypeptide regulators produced from the anterior pituitary gland [29]–[31]. It regulates growth, differentiation, development and metabolism of a vast majority, if not all tissues [32]–[35]. Nevertheless, GH and GH receptor (GHR) are expressed locally in a number of extrapituitary tissues, such as neural tissue, providing autocrine/paracrine activity [36]–[39]. Autocrine GH has regulatory functions in embryonic development, cellular differentiation, and proliferation in neurological, immunological, reproductive, gastrointestinal, skeletal-muscular, respiratory, and cardiovascular systems, and is reported to be involved in the development and metastasis of tumor cells [40], [41].

GH has been reported to be critical in a variety of tissue regeneration, including liver, bone, and muscle [42]–[44]. More strikingly, it elicits therapeutic benefits in rat when used for nerve regeneration [45], [46], and in human burn victims when administered to accelerate wound healing [47], [48]. GH has also been shown to affect apoptotic pathways. For example, human GH decreases apoptosis in neutrophils within blood [49], [50].

However, the role of GH in auditory hair cell regeneration in fishes, or any other organism, has not yet been reported. The purpose of this study was to examine whether exogenous GH promotes hair cell regeneration in the zebrafish inner ear following acoustic trauma, and whether this effect is mediated via cell proliferation and/or apoptosis. In addition, a GH antagonist was used to investigate the role of endogenous GH on cell proliferation in the zebrafish ear post-trauma. The regulatory nature of GH may make it a potential candidate for therapeutic or prophylactic treatment for auditory hair cell loss.

## Results

Five experiments were conducted to examine the effects of GH on the zebrafish inner ear following acoustic trauma. In Experiments 1, 2, and 3, there were three treatments (baseline controls which received no acoustic trauma and no injections, buffer controls which were injected with buffer following acoustic trauma, and GH-treated fish which were injected with exogenous GH following acoustic trauma). The effect of GH on hair cell density, visualized through phalloidin staining of hair cell bundles, was examined in Experiment 1. The effect of GH on cell proliferation, quantified by BrdU labeling, was examined in Experiment 2. GH-induced patterns of apoptosis was examined via TUNEL-labeling in Experiment 3. Fluorescent *in situ* hybridization (FISH) was then used to examine expression patterns of endogenous GH mRNA following sound exposure (Experiment 4). Lastly, a GH antagonist was used to block the effects of endogenous GH on cell proliferation in the zebrafish ear following acoustic trauma (Experiment 5).

### Experiment 1: Effects of growth hormone on hair cell bundle density

**Figure 1:**
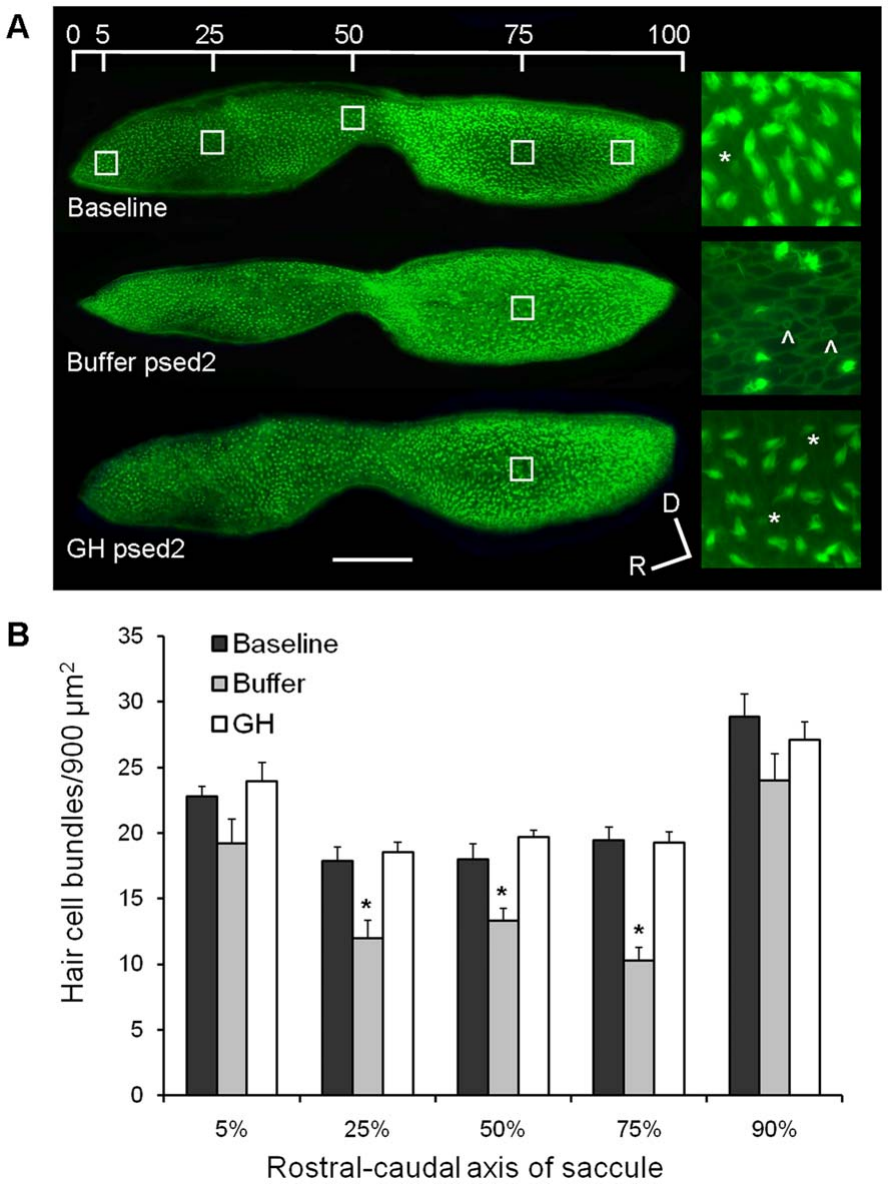
Effect of GH on hair cell bundle density. (A) Phalloidin-labeled saccular epithelia of baseline, buffer-injected, and GH-injected zebrafish at post-sound exposure day 2 (psed2). The upper image shows the five locations of hair cell counts along the rostral-caudal axis of the saccule. The enlarged images to the right of the saccules are representative 100X images of saccules at 75% along the rostral-caudal axis. Scale bar  = 100 µm, D = dorsal, R = rostral, * = presumed newly formed hair cell bundles, ∧ = scar formation characteristic of hair cell loss. (B) Mean (±S.E.) number of hair cell bundles/900 µm^2^ at specified locations along the rostral-caudal axis of the saccule of baseline and buffer- or GH-injected zebrafish. N = 6; * P<0.001.

Saccular hair cell bundle density was reduced in the buffer-injected group 48 hours following sound exposure (post-sound exposure day 2, psed2) compared to baseline and GH-injected fishes (P<0.001; Fig. 1A). Specifically, following acoustic trauma, loss of hair cell bundles was significant at locations of 25%, 50% and 75% along the rostral-caudal axis of the saccules of buffer-injected fish compared to non-sound-exposed baselines (P<0.001; Fig. 1B). The greatest hair cell loss occurred at the 75% location of the caudal saccule. Sound-exposed, GH-injected fish had hair cell bundle densities that did not differ from that of baseline fish at any location in the saccule, suggesting that GH either prevented acoustically-induced hair cell loss or promoted hair cell regeneration quickly following hair cell loss (Fig. 1B).

**Figure 2:**
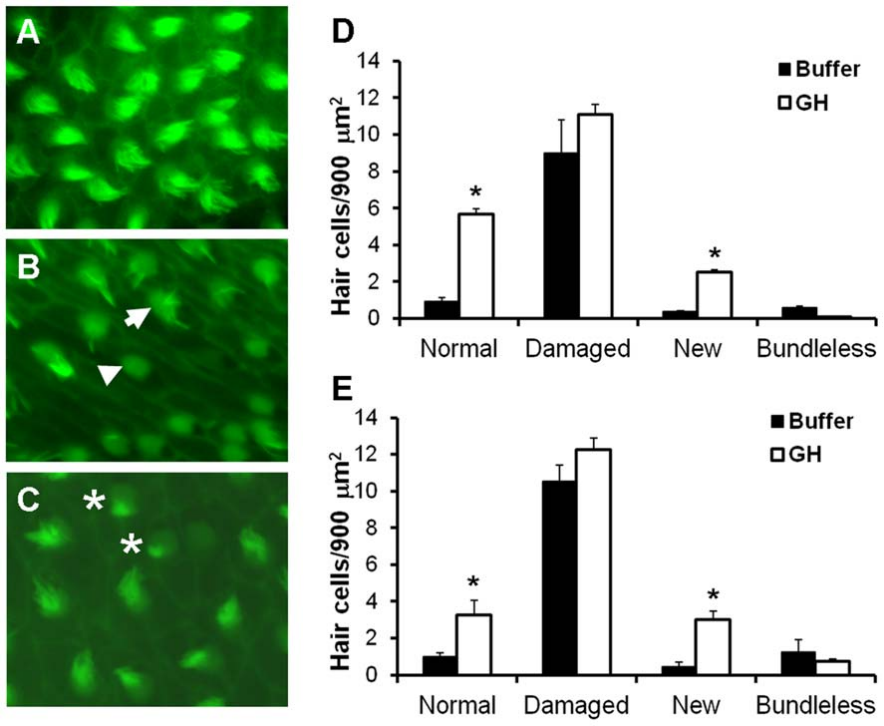
Effect of GH on hair cell type. Phalloidin-labeled saccular epithelia of (A) baseline, (B) buffer-injected, and (C) GH-injected zebrafish at post-sound exposure day 2 (psed2) at 25% along the rostral-caudal axis of the saccule. Arrow = hair cell with missing and splayed stereocilia; triangle = bundleless hair cell, * = presumed newly formed hair bundles. Mean (±S.E.) number of hair cell bundles/900 µm^2^ at (D) 25% and (E) 75% along the rostral-caudal axis of the saccule of buffer- or GH-injected zebrafish. A similar pattern was evident at the 50% rostral-caudal location. N = 6; * P<0.001.

Four types of hair cells were categorized for further analysis. Normal hair cells were defined as those that appeared to have standard numbers and lengths of stereocilia (Fig. 2A). Damaged hair cells were characterized by stereocilia that were few, fractured, or fused (Fig. 2B, arrow). Bundleless hair cells referred to hair cells that had lost all stereocilia, exposing the underlying cuticular plate (Fig. 2B, triangle). Presumed new hair cells exhibited compact, well-ordered, and much shorter stereocilia (Fig. 2C, asterisks). Significantly more normal and new hair cells were found in the growth hormone group compared to the buffer control group at 25%, 50%, and 75% along the rostral-caudal axis of the saccule (P≤0.002; Figs. 2D and 2E).

### Experiment 2: Effects of GH on cell proliferation in the zebrafish ear

**Figure 3:**
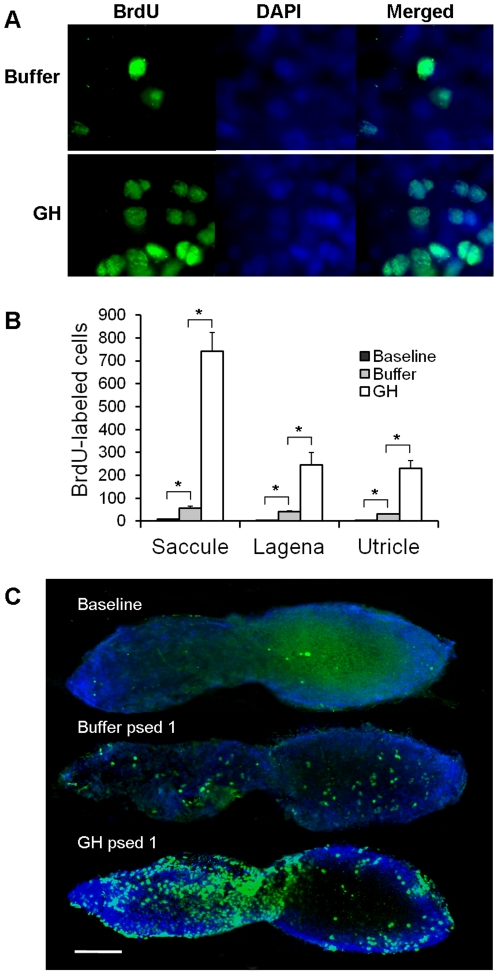
Effect of GH on cell proliferation. (A) 100X images of BrdU and DAPI labeling in the saccules of buffer- and GH-injected zebrafish. (B) Mean (±S.E.) number of BrdU-labeled cells in the saccules, lagenae, and utricles of baseline and buffer- or GH-injected zebrafish. N = 6; * P<0.001. (C) BrdU-labeling in the saccules of baseline, buffer- or GH-injected zebrafish at post-sound exposure day 1 (psed1). Scale bar  = 100 µm. Rostral-caudal orientation is the same as Fig. 1A.

For the cell proliferation assays, BrdU-labeling was co-localized with DAPI in the nuclei of the cells as expected (Fig. 3A). Buffer-injected fish exhibited significantly greater BrdU-labeled cells in the saccule, lagena, and utricle, compared to baseline fish, suggesting that proliferation was a natural response to acoustic trauma (P<0.001; Fig. 3B). Growth hormone injection resulted in much greater proliferation though, with numbers of BrdU-labeled cells being five to ten times greater than buffer-injected controls (P<0.001; Fig. 3B). A few BrdU-labeled cells were found in baseline controls, but these cells were primarily found along the periphery of the macular tissues, whereas the BrdU-labeling in maculae of buffer- and GH-injected fish were much more widespread (Fig. 3C).

### Experiment 3: Effect of GH on apoptosis in the zebrafish ear

**Figure 4:**
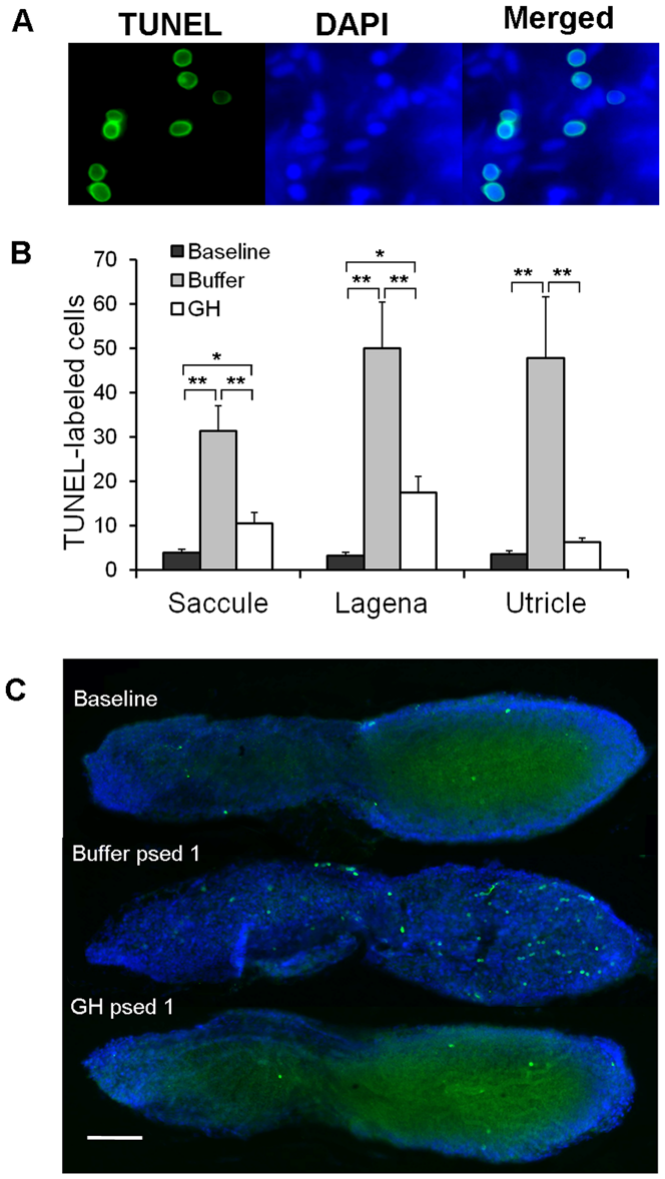
Effect of GH on apoptosis. (A) 100X images of TUNEL and DAPI labeling in the saccule of a buffer-injected zebrafish. (B) Mean (±S.E.) number of TUNEL-labeled cells in the saccules, lagenae, and utricles of baseline and buffer- or GH-injected zebrafish. N = 6; * P<0.01, ** P<0.001. (C) TUNEL-labeling in the saccules of baseline, buffer- or GH-injected zebrafish at post-sound exposure day 1 (psed1). Scale bar  = 100 µm. Rostral-caudal orientation is the same as Fig. 1A.

For the apoptosis assays, TUNEL-labeling was also co-localized with DAPI in the nuclei of the cells (Fig. 4A). Buffer-injected fish had much greater numbers of TUNEL-labeled cells compared to either baselines or GH-injected fish. This was true for all three end organs (saccule, lagena, utricle; P<0.003; Fig. 4B). Sound-exposed, GH-injected fish exhibited more apoptotic cells than baseline controls in the saccule and lagena (P<0.03), but not in the utricle (Fig. 4B). In buffer-injected fish, TUNEL-labeling was generally found to be greater in the caudal region of the saccule compared to the rostral region (Fig. 4C).

### Experiment 4: Localization of GH mRNA following acoustic trauma

**Figure 5:**
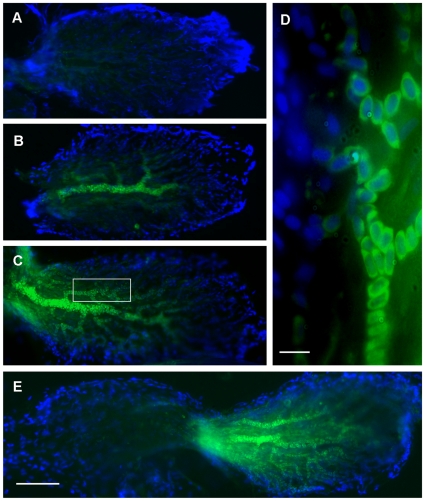
Expression of GH mRNA in zebrafish saccules. Photomicrographs of saccules of zebrafish labeled with probes against GH mRNA via fluorescent *in situ* hybridization (FISH); green = FITC conjugated probe, blue = DAPI. Saccules from (A) a baseline control (not exposed to sound) and (B–E) sound-exposed zebrafish. (D) 100X image of the boxed area in (C), showing that GH mRNA appears to be localized perinuclearly in blood cells. (A–C) show only the caudal portion, while (E) shows a whole saccule. Caudal is to the right as in previous figures. Scale bars (D = 10 µm; E = 100 µm).

Control fish (N = 6) that were not exposed to the acoustic stimulus exhibited minimal labeling for GH mRNA in all end organs (Fig. 5A). In contrast, fish exposed to the sound stimulus (N = 8) exhibited a consistent pattern of labeling that was localized to the caudal region of the saccule (Fig. 5B–E). Specifically, GH mRNA expression was strongest in blood vessels and appeared to be greatest perinuclearly around erythrocytes (Fig. 5D).

Very little positive staining was evident in utricles, but lagenae exhibited intermediate GH mRNA expression that was much more diffuse than found in saccules. Lagenae did not show a localized pattern of expression as saccules did.

### Experiment 5: Effect of a GH antagonist on cell proliferation in the zebrafish ear

**Figure 6:**
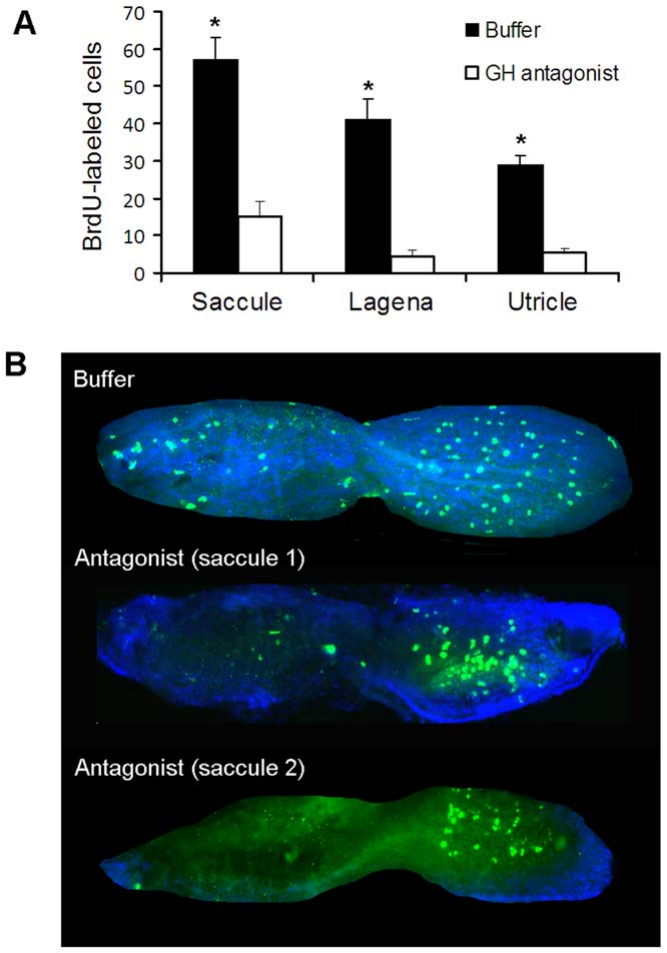
Effect of GH antagonist on cell proliferation. (A) Mean (±S.E.) number of BrdU-labeled cells in the saccules, lagenae, and utricles of buffer- or GH-antagonist-injected zebrafish. N = 6–8; * P<0.001. (B) BrdU-labeling in representative saccules of buffer- or GH-injected (two examples are provided) zebrafish at post-sound exposure day 1 (psed1). Rostral-caudal orientation is the same as Fig. 1A.

While Experiment 2 showed that injection of exogenous GH can promote cell proliferation, injecting a GH antagonist following acoustic trauma allowed us to assess the importance of endogenous GH on cell proliferation in the zebrafish ear. Fish injected with the antagonist had significantly less BrdU-labeled cells one day post-sound exposure compared to buffer-injected controls in all three end organs (Fig. 6A; N = 6–8; P<0.001).

The saccules of buffer-injected fish exhibited BrdU-labeled cells in both rostral and caudal regions, while the saccules of antagonist-treated fish exhibited an interesting pattern- almost no BrdU-labeled cells in the rostral side, but proliferation still occurring centrally in the caudal side (Fig. 6B). While lagenae and utricles of antagonist-injected zebrafish also exhibited a significant decline in BrdU-labeled cells, there was not an obvious localized pattern of this effect on proliferation across the epithelia as was found in saccules.

## Discussion

### Effects of growth hormone on hair cell bundle density

Acoustic trauma can effectively elicit hair cell bundle loss, followed by gradual recovery of hair cells in teleost fishes [17], [26]. Results from the current study suggest that GH may prevent hair cell loss or speed up the process of regeneration. Two days following acoustic trauma, GH-injected fish had saccular hair cell bundle densities that were similar to baseline controls. It is unclear whether this is due to protective effects of GH that might prevent hair cell loss, or whether it is due to the production of new hair cells, but there is evidence for both. Newly synthesized hair cells were those characterized with ordered, compact, and short cilia, which was a subjective judgment as done in previous studies [26]. Exogenous growth hormone promoted this new hair cell bundle formation and more normal-type hair cells after sound exposure, but did not significantly prevent damage to hair cells compared to buffer-injected controls (Fig. 2). The ability of exogenous growth hormone to promote hair cell bundle density supports our hypothesis that endogenous GH plays an important role during normal hair cell regeneration in the zebrafish inner ear.

GH may also play a role in preventing the loss of hair cells following trauma. Although GH did not appear to reduce damage to hair cell stereocilia, our TUNEL data show that it can significantly reduce the apoptosis that normally occurs after acoustic overstimulation (Fig. 4). One potential explanation for this pattern is that hair cells could be damaged by acoustic trauma, but instead of undergoing apoptosis and being ejected out of the epithelia, they could exhibit self-repair of their stereociliary bundles. Further experiments using double-labeling of BrdU and hair cell markers are needed to see if newly-formed hair cell bundles exist in non-mitotic zebrafish hair cells.

One interesting observation was that hair cell bundle damage was generally greatest at 75% along the R-C (rostral-caudal) axis. Similarly, other studies have shown damage to the caudal region of the saccule in response to overexposure to low frequency tones. For example, in zebrafish exposed to a pure tone (100 Hz, 179 dB re 1 µPa RMS) for 36 hours, the area around 75% of R-C axis was the only area that exhibited notable hair cell bundle loss [26]. The reason for the localization of hair cell loss is that the teleost saccule appears to be tonotopically organized, with high and low frequency sounds affecting the rostral and caudal goldfish (*Carrasius auratus*) saccule in a graded manner [51]. One would expect a similar pattern in zebrafish since they are closely related species with virtually identical ears [52]. In the current study, we still observed some consistent loss of hair cell bundles in the zebrafish saccule at the areas around 25% and 50% of R-C axis, but hair cell loss was greatest at the 75% caudal location.

### Effects of GH on cell proliferation in the zebrafish ear

We went further to investigate the cellular mechanisms by which GH is able to promote hair cell regeneration. A significant increase in cellular proliferation was observed in the saccules, lagenae, and utricles of the GH-injected group compared to the buffer group one day post-sound exposure, when the traumatized epithelium was presumably initiating the process of repair. The ability of GH to promote proliferation is not a surprise, as it promotes cellular proliferation in mammary and endometric tissues [53]–[55]. Nevertheless, the potent efficacy of GH on post-trauma proliferation within the zebrafish inner ear is still an encouraging discovery.

Some subtle differences between post-sound exposure proliferation found in this project and that of previous reports were noticed. For example, Schuck et al. [26] found that after 36 hours of sound exposure (100 Hz tone, 179 re 1 µPa), cell proliferation peaked in the zebrafish saccule two days following trauma, with an average of 53 BrdU- positive cells per saccule. In the current study, the average number of BrdU-positive cells was similar (57) to the previous study, but one day earlier post-trauma. This supports the idea that GH speeds up the process of post-trauma cell proliferation. We are currently examining cell proliferation at various time points post-trauma to test this hypothesis.

While it might be suggested that GH simply induces indiscriminate cell proliferation instead of specifically targeting auditory epithelia that has been damaged by acoustic trauma, comparison of our current data with that of a previous study does not support this view. In a previous study, we injected zebrafish that were not exposed to sound with salmon GH. We found that GH induced cell proliferation in the zebrafish ear, although this effect was only significant in the utricle, a vestibular portion of the ear (28). In contrast, in the current study the effect of GH on cell proliferation was greatest in the zebrafish saccule, which is thought to be important for hearing and is known to be damaged by acoustic stimuli (26). Although numbers of BrdU-labeled cells cannot be directly compared between the two studies since different concentrations and sources of GH were used, the fact that GH induced cell proliferation in the saccule of sound-exposed fish but not control fish suggests that acoustically-damaged tissues are more sensitive to GH signaling pathways.

One question that remains to be answered is: what is the fate of the proliferating cells stained positive for BrdU? The answer to this question will shed light on the source of the regenerated hair cells and whether they are the result of direct transdifferentiation or proliferation of surrounding supporting cells. Double staining with BrdU incorporation and parvalbumin or calretinin (both hair cell markers) could be used to examine the expression of hair cell markers on the newly proliferated cells in future experiments.

### Effect of GH on apoptosis in the zebrafish ear

Sound exposure induces apoptosis in the goldfish saccule [17], and in the zebrafish saccule, lagena, and utricle (the current study). However, administration of exogenous GH immediately following sound exposure suppresses apoptosis in the zebrafish inner ear, although not completely compared to baseline controls (Fig. 4). This protective action of GH may be elicited through signaling mechanisms involved in other established neuroprotective pathways. For example, suppression of caspase-3 and AIF (apoptosis inducing factor) expression may contribute to GH-mediated retina cell survival [56]. In the embryonic chick retina, neutralization of endogenous GH leads to undesirable apoptosis [57]. This apoptosis is accompanied with activation of caspase 3 and 9, phosphorylation of AKt (protein kinase B) and TrK (tyrosine kinases), and cleavage of PARP-1 (poly ADP-ribose polymerase I) [57], [58]. These pathways may work together and activate cAMP response element binding protein, followed by initiation of the transcription of anti-apoptotic genes [59].

### Localization of GH mRNA following acoustic trauma

Although a novel finding, it is not altogether surprising that GH mRNA expression would be localized in blood cells. It is known that leukocytes reside in the avian inner ear epithelia and are recruited to areas of hair cell damage [60]–[64]. While the functional role of these leukocytes in the inner ear is currently unknown, it has been hypothesized that macrophages may play a role in wound repair and healing by recognizing and engulfing dying hair cells [62], and that leukocytes secrete growth factors or cytokines that may trigger hair cell regeneration [64].

The cells that expressed GH mRNA in our study did not appear to be leukocytes though, but erythrocytes. The cells were elongated and elliptical with an oval, centrally located nucleus as found in most teleost erythrocytes [65]. They also appeared to make up the large majority of the blood, which would not be the case if they were leukocytes [66]. Although studies have shown that GH can have a significant effect on teleost leukocytes [67]–[68] and human erythrocyte maturation [68], we believe that this is the first report of erythrocytes expressing GH themselves. Radiolabeled GH bound strongly to erythroblasts of sea bream (*Sparus aurata*), showing that teleost red blood cells have receptors for GH [69]. Our *in situ* hybridization data suggests that not only do teleost erythrocytes have the capability of receiving GH signals, but they can also produce paracrine GH signals themselves.

The pattern of much higher levels of GH mRNA being expressed on the caudal portions of the zebrafish saccule than the rostral suggest that the upregulation of GH is being controlled by the local needs of damaged tissues. Since the teleost saccule is tonotopically organized with the caudal portion being more sensitive to low frequency sounds [51], and the caudal portion lost more hair cells than the rostral portion in response to our acoustic stimulus (Fig. 1), it is likely that the inflammation and immune responses associated with wound healing promoted the local upregulation of GH.

### Effect of a GH antagonist on cell proliferation in the zebrafish ear

The zebrafish GH antagonist had a significant effect on cell proliferation in the zebrafish inner ear following acoustic trauma, suggesting that GH is very important, if not necessary, for cell proliferation to occur. A small level of cell proliferation continued to occur following injection with the antagonist, most likely because the concentration of local endogenous GH was greater than that of the antagonist. This may explain the pattern found in the saccules in which proliferation was minimal in the rostral region, but still occurred in the caudal region (Fig. 6B). Our *in situ* hybridization data showed that GH mRNA was much greater in the erythrocytes of the caudal side than the rostral side, and the resulting increased local GH levels likely outcompeted the antagonist for GH binding sites.

### Summary and Future Research

It is reasonable to conclude that growth hormone is a vital regulatory factor for hair cell regeneration in the zebrafish ear following trauma. Since GH-injection reduces the number of saccular cells undergoing apoptosis, and promotes the production of newly formed hair cells, one might expect GH to produce supernumerary hair cells as has been found with other growth factors in neonatal cochleae of rats [70]. This was not found in our study, but as it takes between 7 and 14 days for hair cells to regenerate in the zebrafish saccule following acoustic trauma [26], future experiments at longer time points post-sound exposure are needed to examine the long-term effects of GH on the zebrafish ear and hearing functionality. We have also initiated next generation sequencing experiments to evaluate the gene expression profiles during hair cell regeneration with or without GH stimulation, in the attempt to discover gene candidates involved in GH-mediated hair cell regeneration.

The finding that zebrafish erythrocytes can produce GH is a novel one that needs further examination. If this is true, then what could this mean in terms of GH's potential role in hair cell protection and/or regeneration in the mammalian ear since mammalian erythrocytes are anucleate and thus cannot produce GH? In mammals perhaps leukocytes produce GH signaling instead of erythrocytes. The development of additional probes specific to various types of blood cells would be helpful in examining which specific cell types produce GH, since this would allow double-labeling *in situ* hybridization experiments.

One important advantage of potential GH therapeutics for hair cell regeneration is that it may be injected directly into the cochlea and elicit its pharmacological effects locally in that small compartment. Thus a high localized concentration might be achieved within the cochlea while avoiding systemic side effects of excessive GH such as acromegaly, potential tumorigenesis, electronic and fluid disturbance, intracranial hypertension, and insulin resistance [71]–[73]. At the same time, the simple drug delivery helps get around the obstacles of current stem therapy for hair cell regeneration. For reasons not completely understood, stem cells often fail to incorporate into the cochlea even when they are delivered to the correct location of the ear and have the potential to initiate tumors [7], [74], [75]. Future research examining the effects of localized GH injections into the cochlea of mammalian models is needed to probe the potential for GH therapeutics for deafness in humans.

## Materials and Methods

### Experimental animals

Zebrafish were maintained under standardized conditions and experiments were conducted in accordance with protocols approved by the Institutional Animal Care and Use Committee of Western Kentucky University (Animal Welfare Assurance #A3558-01, Protocol 08-06). Seventy-six adult wildtype zebrafish were used in this study. Fish were obtained from commercial suppliers and maintained in 170-L flow-through aquarium under constant temperature (25°C) and a 12 hours light/12 hours dark cycle.

### Experimental design

In all experiments, groups of at least six fish were exposed to sound (see *Acoustic exposure* below) and then immediately injected intraperitoneally with either carp recombinant growth hormone (GH) or phosphate buffer (PB, 0.1 M, pH 7.4) and then placed in a recovery tank. The results from GH and buffer groups were compared to those from baseline animals (without sound exposure or injection). Apoptosis and cell proliferation were measured at 24 h following acoustic trauma (post-sound exposure day 1, psed1). Hair cell densities were measured at psed2. These time points were chosen since our previous studies with goldfish and zebrafish showed a peak in cell proliferation in the zebrafish saccule at 2 days post-trauma [17], [26] and near-complete hearing recovery occurs between 7 and 14 days post-trauma [17], [76].

### Acoustic exposure

Zebrafish were exposed to a 150 Hz pure tone at a source level of 179 dB re 1 µPa root mean squared (RMS) for 40 h at 25°C in a 19-L sound exposure chamber. The tone was generated by an underwater speaker (University Sound UW-30) and function generator (4017A, B&K Precision) attached to a 5.3 amp/200 watt Audiosource monoblock amplifier. This protocol has resulted in significant hair cell loss in the zebrafish and goldfish saccule in previous studies [26], [51].

### Growth hormone injection

First, carp recombinant growth hormone (GH) powder (ProSpec-Tany Technogen Ltd., Israel) was dissolved into Nanopure water at a final concentration of 0.5 µg/µl. Immediately after sound exposure, fish were placed in water containing a light dose (approximately 10 µg in 1 ml water) of tricaine methanesulfonate (MS-222, Argent, Redmond, WA) for sedation. Then each fish was injected intraperitoneally with approximately 15 µl of GH (20 µg carp GH/gram body mass). Fish were allowed to recover from injection in a small container containing fish tank water. After fish recovered to normal movement, they were put back to their normal aquaria until needed for specific experimental time points. Buffer-injected controls were handled the same way except that they were injected with approximately 15 µl of PB (0.1M, pH 7.4).

### Quantification of hair cells bundles

Two days after sound exposure and the injection (psed2), fish (n = 6 per group) were euthanized with an overdose of tricaine methanesulfonate (MS-222, Argent, Redmond, WA) at a concentration of approximately 250 mg/L. The heads were removed and fixed in 4% paraformaldehyde at 4°C overnight. Then they were washed 3×10 minutes with 0.1 M pH 7.4 PB, and the inner ear maculae (saccules, lagenae, and utricles) were dissected out of the head and excess tissue trimmed. All tissue was incubated in concavity wells with 1∶100 fluorescein phalloidin (F432, Invitrogen) in phosphate buffer (PB) at room temperature in a dark box for 1 h. After incubation, end organs were placed on glass slides mounted with Prolong Gold Antifade DAPI mount reagent (P36931, Invitrogen) and then cover-slipped. Low power images (10X objective) of the saccule, lagenae and utricle were viewed under FITC and DAPI filters of an Zeiss Axioplan 2 epifluorescent microscope and photographed with an AxioCam MRm camera. Hair cell bundle counts were obtained from 5 preselected 30 µm×30 µm locations along the rostral-caudal axis of the saccule (5%, 25%, 50%, 75%, and 90%; Fig. 1A), as was done in a previous study [26]. These locations were chosen because acoustic trauma tends to cause hair cell loss in the central, interior portion of the saccule, and these five locations represent a wide range in hair cell density [26] and sensitivity to frequency [51] along the rostral-caudal axis of the zebrafish saccule. At each location, 30 µm×30 µm digital boxes were placed over 100X objective microscopic images in Photoshop. Under the FITC filter, the phalloidin-labeled hair cell stereocilia fluoresced green which allowed easy quantification of their numbers. DAPI staining (blue color) allowed the visualizing of cell nuclei. Normal hair cells were quantified as whole hair cell bundles with intact, long stereocillia; damaged hair cell bundles had broken, disordered, or sparse stereocilia; presumed newly formed hair cell bundles were those defined as having compact, well-ordered, and much shorter than normal hair cell bundles; bundleless hair cells lacked any stereocilia, exposing the underlying cuticular plates.

In addition, the other two end organs of zebrafish inner ears besides the saccule, the lagena and utricle, were also collected and stained with phalloidin. No remarkable loss of hair cells was obvious, so they were not subjected to formal analysis due to the difficulty of defining the specific areas for counting.

### Determination of cell proliferation

Following sound exposure and injection of GH or buffer, fish were allowed to recover for 8 h in a recovery tank and then were used for BrdU incorporation assay with the standard protocol of the Amersham Cell Proliferation Kit (RPN20LR, GE Healthcare). Bromodeoxyuridine (BrdU) is a synthetic thymidine analog and can be incorporated into cellular DNA during S-phase, thus allowing to detection of cell proliferation. Fish (n = 6 per group) were injected intraperitoneally with the BrdU and then placed in a separate tank. After 16 h (at psed1), fish were euthanized with an overdose of MS-222 and then their heads were removed and their inner ears were immediately dissected out and fixed in 4% paraformaldehyde for 1 h. After washing with 0.1 M PB, the intact saccules, lagenae, and utricles were trimmed of excess tissue and placed on adhesive poly-L-Lysine coated slides and then incubated for 1 h at room temperature in mouse monoclonal anti-BrdU antibody. After washing in 0.1 M PB 3×5 minutes again, tissues were incubated with 1∶200 Alexa Fluor 488-conjugated goat anti-mouse IgG (A11001, Invitrogen) for 1 h at room temperature in a dark box. Finally, the tissues were washed 3×5 min with 0.1 M PB and these end organs were then mounted with Prolong Gold Antifade reagent with DAPI (P36931, Invitrogen) and then cover-slipped. Low (10X objective) and high (100X objective) power images of the saccule, lagenae and utricle were viewed under FITC and DAPI filter of Zeiss Axioplan 2 epifluorescent microscope and images were photographed by an AxioCam MRm camera, with image contrast adjusted for best visualization of labeled cells. The quantitative evaluation of cell proliferation was determined by counting Alexa Fluor 488-labeled cells in both (left and right) whole saccules, laganae, and utricles of each individual fish.

### Determination of cell death

In order to detect apoptosis at psed1, fish were euthanized with an overdose of MS-222 (n = 6 per group), and the heads were removed. Inner ears were dissected out and immediately fixed in 4% paraformaldehyde. A terminal deoxynucleotidyl transferase dUTP nick end labeling (TUNEL) assay was used to label cells with DNA fragmentation indicative of apoptosis. After washing with 0.1 M PB, the intact saccules, lagenae, and utricles were then trimmed and placed on adhesive poly L-Lysine coated slides and following the standard instruction of ApopTag Fluorescein In Situ Apoptosis Detection Kit (S7710, Millipore). Tissues were post-fixed in pre-chilled ethanol:acetic acid 2∶1 for 6 min at −20°C and then washed 3×5 min with 0.1 M PB. Then equilibration buffer was applied directly on the slide and incubated at room temperature for at least 10 s. The buffer was rinsed and TdT (terminal deoxyribonucleotidyl transfer) enzyme was added on the slide and incubated in a humidified incubator at 37°C for 1 h. Stop/Wash Buffer was applied, agitated for 15 seconds, and then incubated at room temperature for 10 min. After rinsing 3×5 min by 0.1 M PB, Anti-Digoxingenin Conjugate Fluorescein was added to the slides and incubated in a humidified container in dark at room temperature for 1 h. The slides were washed with 0.1 M PB 3×5 min again and mounted with the Prolong Gold Antifade reagent with DAPI and then cover-slipped. Labeled cells were counted manually under a Zeiss compound microscope at 10X and 100X magnification.

### Zebrafish GH mRNA in situ hybridization

Zebrafish (N = 8) were subjected to sound exposure as described above. Inner ears were taken out and fixed in DEPC-treated 4% PFA for 1 h at RT. Auditory sensory epithelium was dissected out and placed on adhesive microslides (Newcomer Supply, catalog 5110). After washing, tissues were permeabilized for 30 min at 37°C with TE buffer (100 mM Tris-HCl, 50 mM EDTA, pH 8.0) containing 10 µg/ml RNase free Proteinase K (Qiagene, catalog 19131). Tissues were post-fixed for 5 min at 4°C with DEPC-treated PB buffer containing 4% paraformaldehyde. After washing, tissues were acetylated by incubating with 0.1 M triethanolamine (TEA) buffer, pH 8.0, containing 0.25% (v/v) acetic anhydride for 10 min on a rocking platform. Slides were incubated at 37°C for at least 10 min with prehybridization buffer, which was 4X SSC (saline sodium citrate buffer) containing 50% (v/v) deionized formamide, at 37°C for 2 h. 20 µl of hybridization buffer containing the oligonucleotide probe (1∶9 =  probe:prehybridization buffer) was added to samples and incubated at 37°C overnight in a humid chamber. The oligonucleotide probe for zebrafish GH mRNA was costumer synthesized by Creative Biomart (Shirley, NY, USA) with the sequence of 5′GCTGATGCCCATTTTCAGGTCCGCCAGTTTCTC3′ and conjugated with FITC. After washing, zebrafish GH mRNA *in situ* hybridization was visualized under fluorescence microscopy. The expression patterns of GH mRNA were compared between sound-exposed zebrafish (N = 8) and non-sound-exposed zebrafish (N = 5).

### Zebrafish GH antagonist

A mutant zebrafish GH peptide, which retains receptor binding ability but no intrinsic GH activity, was customer synthesized by ProSpec-Tany Technogen Ltd. (Israel). Considering the competitive antagonistic property of the mutant, a relatively high dose (40 µg/gram body mass) of the peptide was injected intraperitoneally into a group of fish (N = 6) at the end of the sound exposure. BrdU assay was then performed one day after sound exposure as described above, and compared to BrdU labeling in buffer-injected controls (N = 8).

### Statistical analysis

Analysis of variance (ANOVA) was used to test for differences between treatments (baseline, buffer-injected, and GH-injected) for all of the endpoints quantified. Separate ANOVAs were done for each location along the rostral-caudal axis for hair cell bundle counts, for each hair cell morphotype in each location, and for each end organ (saccule, lagena, utricle) for BrdU- and TUNEL-labeled cells.
